# Effect of exercise and morphine on psychological and physical dependencies, BDNF and TrkB gene expression in rat’s hippocampus

**DOI:** 10.12669/pjms.333.12342

**Published:** 2017

**Authors:** Mojtaba Naghshvarian, Mohammad-Reza Zarrindast, Shahram Ejtemaei Mehr, Mohammad Mehdi Ommati, Seyedeh Fatemeh Sajjadi

**Affiliations:** 1 Mojtaba Naghshvarian, Department of Pharmacology, School of Medicine, Tehran University of Medical Sciences, Tehran, Iran; 2 Mohammad-Reza Zarrindast, Department of Pharmacology, School of Medicine, Tehran University of Medical Sciences, Tehran, Iran; 3 Shahram Ejtemaei Mehr, Department of Pharmacology, School of Medicine, Tehran University of Medical Sciences, Tehran, Iran; 4 Mohammad Mehdi Ommati, Department of Animal Sciences, School of Agriculture, Shiraz University, Shiraz, Iran; 5 Seyedeh Fatemeh Sajjadi, Department of Psychology, University of Otago, Dunedin, New Zealand

**Keywords:** Physical activity, Morphine, Abstinence syndrome, Psychological dependency, BDNF, TrkB Hippocampus, Rat

## Abstract

**Objectives:**

To compare the effect of exercise and morphine on abstinence syndrome and hippocampal gene expression in rat model.

**Methods:**

Thirty adult male rats were exposed to voluntary wheel exercise (low, medium, high) for 28 days. The subjects entered Conditioned Place Preference (CPP) apparatus and experienced morphine (low, medium, high) CPP and followed by naloxone test. Correlation between exercise level, morphine injection, concurrent morphine administration and exercise with morphine CPP, BDNF and TrkB genes was determined. Rats were euthanized, decapitated and the hippocampus was removed. The expression of BDNF and TrkB genes were evaluated by real time PCR.

**Results:**

Active rats ran an average of 839.18 m/d. A significant (P<0.001) correlation between exercise level, morphine injection, concurrent morphine administration and exercise with morphine CPP and BDNFand TrKB gene expressions was found.

**Conclusion:**

Voluntary exercise in different levels potentiates the brain rewarding system, CPP scale, and hippocampal BDNF and TrKB expressions. High range of voluntary exercise demonstrated an increase in the likelihood of developing addictive and drug-seeking behavior.

## INTRODUCTION

Drug addiction is considered as a serious and prolonged relapsing brain disorder described by pathological and compulsive drug seeking tendencies and drug abuse, paired with the exhibiting negative emotional states in the case of drug abstinence and negative outcomes for both drug abuser and their families.^1^ There is evidence to suggest that rewarding behaviors are more likely to initiate compulsion and eventually become addictive. An aspect especially worthy of note is that the brain is not able to recognize emotions of reward caused by a chemical or an experience (e.g., exercise).^2^ Exercise also affect the central dopaminergic, noradrenergic, and serotonergic systems through mechanisms, including neurogenesis, mood enhancement, and endorphin secretion.^3^,^4^

Conditioned Place Preference (CPP) is defined as repeated pairing of a specific setting with morphine. When paired stimulus is rewarding and reinforcing to the animal, the preference for the paired setting is enhanced.^5^ It has been demonstrated that some forms of exercise may diminish CPP,^6^ but the effect of prolonged and voluntary exercise on CPP to morphine has not still been determined. Brain-derived neurotrophic factor (BDNF) is characterized by a neurotrophin that exerts support of neuronal survival, differentiation, and connectivity which can have an impact on substance dependence.^7^

It has been found that voluntary exercise is more influential in motor recovery and on enhanced BDNF level in the hippocampus than forced running.^8^ Exercise can increase the availability of BDNF, in certain areas of the brain including the hippocampus and cortex.^7^ Given that the most noted expression of BDNF and its robust correlated receptor, TrkB (Thyrozine Kinase Type-B), was found in brain tissues, such as hippocampus and cerebellum indicating that BDNF may modify neuronal plasticity in adult animals;^9^ more importantly BDNF operates upon the reward system of the brain, which has a main role in drug dependency;^10^ thus BDNF is considered to be a bettercandidate for mediating the long-term benefits of exercise on the brain^7^ and a candidate locus correlation with drug dependence.^10^ Based on this body of prior works, the effect of physical activity and morphine on psychological and physical dependencies and BDNF and TrkB expression in rat’s hippocampus were assessed.

## METHODS

Thirty adult male Sprague–Dawley rats (275-300 g, aged 60 days) were provided from Comparative and Experimental Medicine Center, Shiraz, Iran. Animals were randomly divided into three exercise and two control groups (each groups consisted 6 animals). The sample were individually housed under standard condition (22.0±2°C, 12L: 12D schedule; lights off at 7:00 a.m. and on at 7:00 pm) and had access to food and water ad-libitum. All experiments took place during the light phase. Animal received human care and were handled according to the animal handling protocol approved by a local committee at Shiraz University of Medical Sciences (IR.Sums.Rec1395-.S1218).

### Drugs

Morphine sulphate and naloxone hydrochloride (Darou-Paksh Co., Iran)were used in this experiment. The drug volume or vehicle was calculated based on rat’s body weight and was injected intraperitoneally. In this study, insulin syringe (12 ml) was used for injections.

### Apparatus

The cages that the animals were housed in were 43×23×15 cm and had custom-made wheels (circumference of 1.07 meters) interfaced with a digital micro-switch counter to record the number of wheel revolutions twice a day. Sedentary rats were housed in cages without running wheels to identify them from those described as exercising rats (n=6).

### The Conditioned Place Preference (CPP)

The test box was composed of two equal-sized wooden compartment and Guillotine-style doors to allow accessibility between boxes and the central corridor that stood up for this procedure. The vertical black strip on the white walls is the indicator of the right room and its floor was covered with textured cardboard. The left room with the indicator of black horizontal strip on the white walls had the floor made of clear plastic. The central corridor was composed of wooden walls and floor and had two doors into both rooms through which the rat could commute. Through the conditioning processes, these doors were completely blocked and the test box was placed under conditions of faint illumination (40 Lux) in the silent laboratory.^6^ The box was equipped with a webcam to allow experimenters to observe all the experiment procedure from a separate room.

### Chronic Daily Voluntary Running

In first phase, five days after arrival at the center, the rats were assigned randomly to individual cages with and without wheel as exercise and sedentary group, respectively. In 2^nd^ phase, for about four weeks, rats in the exercise condition had continuous access to wheels set up in their cages. The number of wheel revolutions for each rat was registered twice a day. Based on rat’s running tendency, animals were divided into three categories (low, medium and high exercise groups) and different doses of morphine were used to study its effects more accurately. CPP procedure included three phases as follows:

### (i) Preconditioning phase

After four weeks of wheel running, animals were placed in central corridor with the open guillotine doors to allow access to the entire apparatus for 30 minutes. Video recordings were reviewed and time spent in each chamber was documented.

### (ii) In conditioning phase (Five days)

The rats received drug in one of the two chambers (right or left) in a counter balanced fashion (the ‘unbiased’ procedure). Each animal was conditioned to associate morphine with the chamber of the CPP apparatus that was initially non-preferred or saline placing in the initially preferred chamber of the CPP apparatus. All to-be conditioned, animals were injected intra-peritoneally with either saline or morphine (in 0 mg/kg morphine, n=6; 2.5 mg/kg morphine, n=6; 7.5 mg/kg morphine, n=6; or 10 mg/kg morphine, n=6).^6^ Morphine sulfate or the equal volume of isotonic saline solution was injected twice a day in the morning and afternoon with at least 6 hours interval. Five minutes after injections, the rats were placed in the chambers for 45 minute.

### (iii) In post-conditioning phase

**Fig.1:**
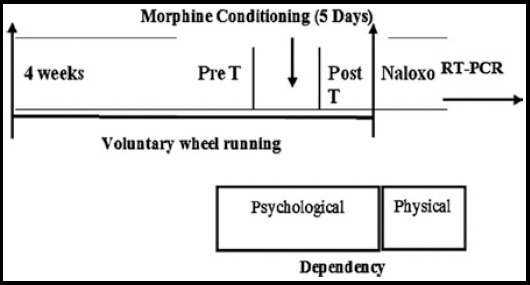
Voluntary wheel running and conditioned place preference timeline.

The day after 5 days of conditioning trial, a CPP test was undertaken. The rats were placed in the central corridor of CPP apparatus with the Guillotine doors raised for 30 min. The amount of time spent in each room was recorded. No injections were given. CPP score was calculated by subtracting the amount of time)t_1_) spent in the unconditioned chamber (morphine paired) on the initial assessment day from the amount of time (t_2_)spent in that chamber during the test day. The entire phases of the experiment procedure were delineated in Fig.1. Chronic wheel running began four weeks prior to a conditioned place preference test for morphine. Exercise was continued throughout the CPP paradigm, until the final CPP. Finally, naloxone tests were done till all rats were sacrificed, stored at -70°C, and prepared for real time PCR.

### Withdrawal Rating Scale

The day following CPP test, rats received naloxone hydrochloride (4 mg per kg)^11^ and after naloxone injection or vehicle, theywere taken into plexiglass boxes and observed blindly for 30 minutes. Withdrawal signs were scored on the basis of modified version of the Gellert–Holtzman scale (1978).^12^ The absence or presences of the consequent opioid withdrawal symptoms were weight loss, irritability, jump, diarrhea, ptosis, and penile erection/ejaculation.

### BDNF and TrkB Expression in Hipocampous

Approximately 24 hours after testing, the rats were euthanized and the hippocampus was removed and stored at −70°C until real time PCR was conducted as described by Livak KJ, Schmittgen (2001) for RNA isolation, cDNA synthesis and amplification reactions.^13^ Line Gene K Thermal Cycler Software (Bioer Technology Co, Hangzhou, China) was used for CT calculation. All amplification reactions were repeated twice under identical conditions and the values of relative mRNA expression were means of three independent experiment±SD. The primer sequence used in polymerase chain reaction was demonstrated in Table-I.

**Table-I:** Primer sequence used in polymerase chain reaction.

*Genes Name*	*Orientation*	*Sense 5^’^-3^’^ sequence*	*Product length (bp)*
GAPDH	Forward	CTCCCATTCTTCCACCTTTG	185
	Reverse	CTTGCTCTCAGTATCCTTGC	
BDNF	Forward	AGTGGCTGGCTCTCATACC	132
	Reverse	TGTCTTGTCTTGTCTTGTCCTG	
TrKB	Forward	CCAGCACATTCTTCTTCATC	194
	Reverse	GGAGCAGGAGTAAGTAAGC	

### Statistical analysis

All statistical analysis was conducted using Statistical Package for the Social Science (SPSS) Version 22. The results of two-way ANOVA(Factors: morphine dose and exercise) was performed to evaluate the effects of exercise, morphine and the morphine exercise interaction on condition place preference (CPP), withdrawal rate, BDNF and TrKB expression in hippocampus.

## RESULTS

### Running Rates and Wheel Running Trend

**Fig.2:**
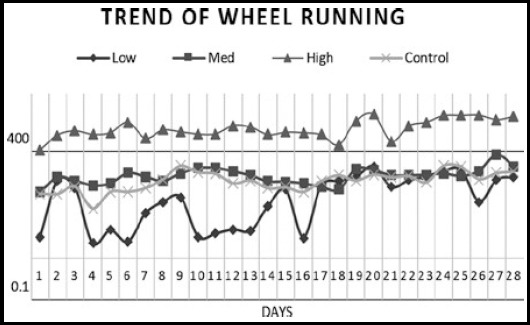
Trend of wheel running for 28 days/group.

Exercising rats ran an average distance of 839.18 m/day, with a range across rats from 11.77 to 10832 m/day. Rats with low exercise ran in a range of 0-80 rev/day (38.466 m/day), in medium exercise ran from 80 to160 (107.59 m/day) and in high exercise ran more than 160 rev/day (1601.94 m/day). As can be seen in Fig.2, there was a tendency for rats in low, medium and high exercise groups to increase their daily run rates over 28 days of voluntary wheel running.

### Exercise Effects on CPP, Withdrawal, BDNF and TrKB

The effects of exercise, morphine and the morphine exercise interaction on condition place preference (CPP), withdrawal rate, BDNF and TrKBexpressions in hippocampus were presented in Table-II. A significant effect of exercise level (F=71.9; df=2; *p*<0.001), morphine injection (F=106.5; df=3; *p*<0.001) and the morphine exercise interaction (F=858.6; df = 6; *p*<0.001) was noticed on CPP. In contrast, the results examining the effect of exercise (F=1.75; df=2; *p*=0.21), morphine (F=3.03; df=3; *P*=0.07) and the concurrent effect of morphine and exercise (F=0.99; df=6; *p*=6.32) on withdrawal indicated no significant effect.

**Table-II:** Tests between subjects and variables.

*Dependent Variables*	*Source*	*Sum of square*	*df*	*Mean Square*	*F*	*Sig.*
CPP	Exercise	215.7	2	107.86	71.99	P<0.001
	Morphine	478.77	3	159.59	106.5	P<0.001
	Interaction of morphine and exercise	858.69	6	143.115	95.52	P<0.001
Withdrawal	Exercise	22.34	2	11.17	1.75	P= 0.21
	Morphine	57.87	3	19.29	3.03	P=0.07
	Interaction of morphine and exercise	37.93	6	6.32	0.99	P=0.47
BDNF	Exercise	25.57	2	12.78	26.30	P<0.001
	Morphine	61.81	3	20.60	42.38	P<0.001
	Interaction of morphine and exercise	41.36	6	6.89	14.18	P<0.001
TrKB	Exercise	4.52	2	2.26	26.61	P<0.001
	Morphine	8.47	3	2.82	33.22	P<0.001
	Interaction of morphine and exercise	8.14	6	1.35	15.96	P<0.001

**Fig.3:**
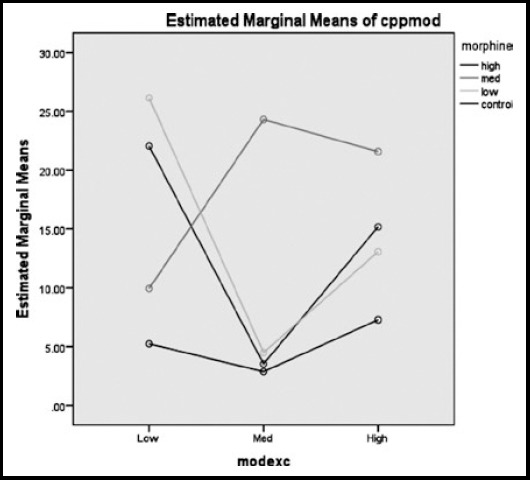
The estimated marginal means of CPP mode, considering three different doses (low, medium, high) of morphine and exercise.

Additionally, exercise (F=26.30; df=2; *p*<0.001), morphine (F=42.38; df = 3; P<0.001) and the concurrent effect of morphine and exercise (F=14.18; df=6; *p*<0.001) had a significant positive effect on BDNF. A significant main effect for exercise (F=26.61; df=2; *p*<0.001), morphine (F=33.22; df=3; *p*<0.001) and the concurrent effect of morphine and exercise (F=15.96; df=6; *p*<0.001) and TrKB was noted. The estimated marginal means of CPP mode (Fig.3) indicated that those rats in low level of exercise and morphine reach the highest morphine CPP score, suggesting that these rats preferred to stay in morphine paired room. The rats in medium level of exercise and morphine intake showed the second highest morphine CPP score, and the rats in low and high levels of exercise which received the high and medium doses of morphine respectively, showed almost the same morphine CPP mean score.

**Fig.4:**
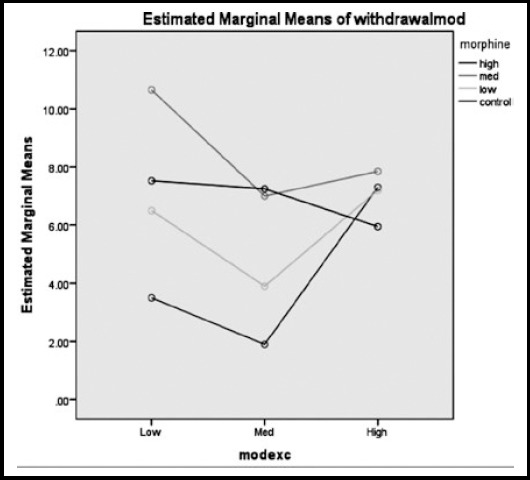
The estimated marginal means of Withdrawal mode, considering three different doses (low, medium, high) of morphine and exercise.

Regarding the data presented in Fig.4, the interaction of medium levels of exercise and high dose of morphine could lead to the minimum mean score of withdrawal, while low level of exercise and medium level of morphine injection caused the maximum withdrawal mean score in rats. Although low levels of exercise did not have a significant effect on withdrawal signs; medium levels of exercise could reduce physical withdrawal signs of morphine. High levels of exercise with the interaction of different doses of morphine had also a similar effect on physical withdrawal signs.

**Fig.5:**
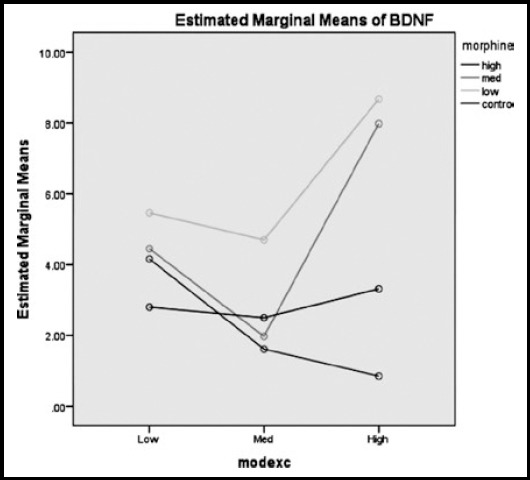
The estimated marginal means of BDNF, considering three different doses (low, medium, high) of morphine and exercise.

**Fig.6:**
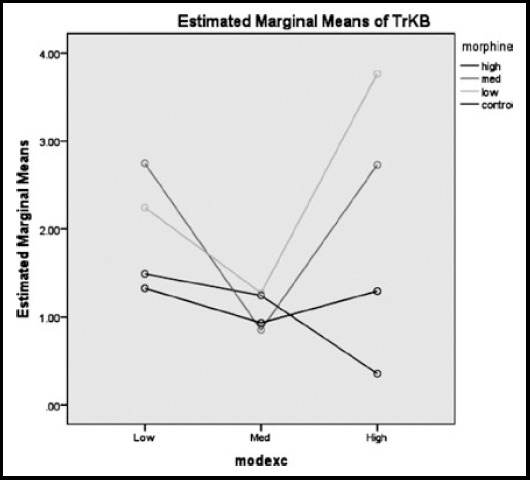
The estimated marginal means of TrKB, considering three different doses (low, medium, high) of morphine and exercise.

As can be seen in Fig.5, those rats in high levels of exercise and low and medium morphine dose acquired the two highest mean score of BDNF, respectively. Whereas, those in high levels of exercise and high dose of morphine injection showed the lowest mean score in BDNF. Considering the Fig.6, the highest mean score of TrKB was allocated to the rats in high level of exercise and low morphine intake and the lowest mean score of TrKB was allotted to those animals with both high level of exercise and morphine dose. The mean score of the rats in low and high level of exercise with medium amounts of morphine were almost the same.

## DISCUSSION

Our findings suggest that running is rewarding for the animals and voluntary wheel running is a pre-clinical model for exercisein humans. More specifically, physical activity and exercise were demonstrated to trigger the substance abuse.^14^,^15^ Prolonged exercise was shown to continuously release endogenous opioid peptides and then a decrease in sensitivity to morphine and other mu opioid agonists.^16^ Persistent running wheel activities was used in rats to enhance baseline pain sensitivity while diminishing the antinociceptive potency of opiate drugs.^16^

Numerous studies have demonstrated that morphine and forced exercise had a significant effects on CPP.^17^,^18^ Using animal models, it was demonstrated that voluntary exercise is a factor which protect individuals from memory and cognition deficit occurring with aging,^19^ inducing the development of cerebellum, motor cortex and capillaries in the rat’s hippocampus,^20^ enhancing learning acquisition and memory retention,^21^ and reducing brain insult.^22^

These findings revealed that different levels of voluntary exercise can reinforce morphine CPP. This may provide further insight into the role of voluntary exercise and suggest that the negative effect of exercise on morphine CPP reported by prior studies may be related to the forced nature of exercise. Voluntary running allows animals to choose how much to run and avoid confounding variables such as the stress induced by forced running.^23^

Such findings fit well with Greenwood and colleague findings about the rewarding properties of voluntary exercise measured by CPP.^24^ The results of our study are in the same line with the research done by Eisenstein and Holmes,^6^ however, what seems meanwhile important is that we reported increased CPP scores, which is an indicator for increased time spent in morphine paired chamber, demonstrating that chronic voluntary exercise increased associative learning in morphine-CPP.

Consistently with existing literature,^21^ the results of our study did not find any significant effect of voluntary exercise on decreasing the physical withdrawal signs of morphine in rats. Although the neurobiological mechanism underlying the withdrawal signs decrement as a consequence of voluntary exercise is yet unclear, a possible explanation for this may be related to the development of cross-tolerance phenomenon. However, the same effect mechanism of morphine and exercise on neurotransmitters and neural receptors (effect on endorphins and endorphin receptors) as well as changes in potency and sensitivity of morphine as a consequence of voluntary exercise^21^ may lead to reducing physical withdrawal signs in our exercising rats. Moreover, this phenomenon is less likely to indicate its effect on physical withdrawal than psychological symptoms like CPP.

Our findings are similar to many studies^23^,^25^,^26^ providing evidence that exercise can increase the BDNF and TrKB levels in hippocampus of morphine-dependent rats. Identical to previous studies,^27^,^28^ our study supported that BDNF acts via the TrKB receptor is an important mediator between exercise and neural functions and play a key role in cell survival, neurite outgrowth, function of adult neurons, differentiation of developing neurons, learning and memory for morphine administration,^29^ and protects against neurodegenerative disorders.^25^

It was shown that repeated morphine administration during the acquisition of morphine CPP was correlated with increased BDNF expression.^29^ The low doses of morphine also can increase BDNF level, whereas the high doses of morphine will have toxic effect on the expression and function of BDNF. TrKB (as the receptor of BDNF) also increases by increasing the levels of exercise and doses of morphine. If neurotrophic factors, including BDNF and TrKB, fall below a certain level, the neurons vulnerability will increase, or conversely, by increasing the levels of these factors, the protection of neurons will raise.^30^

The positive effect of exercise on the brain is mediated by altering BDNF expression. The increased availability of BDNF protected vulnerable cells and caused decrease in neural loss.^30^ It has been shown that adrenal hormone and stress decreased the level of BDNF expression and made neurons more vulnerable, and gave that exercise reduced stress level, and this may explain the up-regulation of BDNF following exercise.^30^

### Limitations of the Study

First, due to ethnical considerations, we minimized our population as little as possible. Second, There was the potential loss of rats after injection of morphine and during testing. The third limitation was related to generalizing the results from animal species to the human population. Aditionally, different kinds of animals, genetic and strain; as well as gender difference (in this study only male rats were used) should be considered as limitations. Our findings should be verified with a larger sample size. further studies need to be done whether forced exercise has similar effects on morphine CPP, BDNF and TrKB as well as withdrawal signs.

## CONCLUSION

Collectively, our results demonstrated that chronic voluntary exercise can increase morphine dependency and then lead to craving increment of drug-seeking behavior.
